# How dietary patterns affect left ventricular structure, function and remodelling: evidence from the Kardiovize Brno 2030 study

**DOI:** 10.1038/s41598-019-55529-5

**Published:** 2019-12-16

**Authors:** Andrea Maugeri, Jana Hruskova, Juraj Jakubik, Ota Hlinomaz, Jose R. Medina-Inojosa, Martina Barchitta, Antonella Agodi, Manlio Vinciguerra

**Affiliations:** 1 Department of Medical and Surgical Sciences and Advanced Technologies “GF Ingrassia”, University of Catania, Catania, Italy; 2 International Clinical Research Center, St’Anne University Hospital, Brno, Czech Republic; 3 Division of Preventive Cardiology, Department of Cardiovascular Medicine, Mayo Clinic, Rochester, MN USA

**Keywords:** Cardiac hypertrophy, Obesity, Epidemiology

## Abstract

Little is still known about the effect of dietary patterns on left ventricular hypertrophy (LVH). Here, we derived dietary patterns by principal component analysis (PCA) and evaluated their association with LV structure, function, and remodelling. Our cross-sectional study included 438 members (aged 25–65 years; 59.1% women) of the Kardiovize Brno 2030 with no history of cardiovascular disease. Two dietary patterns were derived using PCA, namely prudent and western. Primary outcomes were echocardiographic parameters and LV geometric patterns, such as concentric LV remodelling (cLVR), concentric LVH (cLVH), and eccentric LVH (eLVH). Interestingly, participants with high adherence to the prudent dietary pattern had decreased odds of cLVH after adjustment for socio-demographic, clinical and behavioral covariates (OR = 0.24, 95% CI = 0.08–0.88; p = 0.031). By contrast, several echocardiographic parameters increased with increasing adherence to the western dietary pattern, which resulted in higher odds of cLVH among participants with high adherence (OR = 5.38, 95% CI = 1.17–23.58; p = 0.035). Although our findings may have an immediate relevance for public-health strategies, further large-size prospective studies should be encouraged to better understand the observed association and their causality.

## Introduction

Several lines of evidence have shown that subclinical changes in left ventricular (LV) structure and function often precede symptomatic heart failure, and that LV remodelling (LVR) commonly follows cardiovascular events, such as myocardial infarction, idiopathic dilated cardiomyopathy, and volume or pressure overload^1–3^. The classification of LV geometry - proposed by Ganau and colleagues in 1992 - distinguished three abnormal LV geometric patterns based primarily on LV mass indexed to height^2.7^ (LVMI) and relative wall thickness (RWT) parameters^4^. Eccentric LV hypertrophy (eLVH) - characterized by the enlargement of cavity without changes in wall thickness - is mainly induced by volume overload. Instead, concentric LV remodelling (cLVR) and hypertrophy (cLVH) are characterized by an increase in thickness of the myocardium without a corresponding increase in LV size. cLVR and cLVH are traditionally considered as unfavorable LV adaptation to pressure overload, such as hypertension^5,6^. In general, these LV geometric patterns are associated with cardiovascular disease (CVD) and all-cause mortality^4^, while elevated LV mass (LVM) and systolic disfunction are risk factors for heart failure in asymptomatic individuals^7^. Obesity and hypertension - two of the major threats to public health that have been consistently and strongly associated with CVD incidence and mortality^8,9^ - are the main determinants of LVR, and there is consensus that their coexistence has an additive or even synergistic deleterious effect on LVR^6,10,11^. Thus, acting on risk factors for LVR might be helpful in reducing the burden of CVD worldwide.

In 2014, we established the Kardiovize Brno 2030 study, a prospective cohort recruited from the urban population of Brno (Czech Republic), which aims to investigate traditional and novel risk factors for CVD outcomes^6,12–16^. In this cohort, we have already demonstrated, on the one hand, the harmful effect of a western dietary pattern on blood pressure, fasting glucose and triglycerides, on the other hand, the protective effect of a diet rich in cereals, fish, fruit and vegetables against obesity^12^. Previous studies also demonstrated favorable effects of prudent dietary patterns, such as the Mediterranean diet and the Dietary Approaches to Stop Hypertension (DASH) diet, on LV structure and function^17–19^. By contrast, a dietary pattern rich in processed foods and poor in fruit and vegetables might be associated with metabolic dysfunction and aberrant LV function^20^. Although the identification of risk factors for LVR could have immediate relevance for public-health strategies, little is still known about the effect of dietary patterns in patients who are free of CVD. To our knowledge, no evidence exists about the association between a posteriori dietary patterns and LVH and/or remodelling. Here, we used the principal component analysis (PCA) to identify dietary patterns that maximally reflected dietary habits in a subsample of the Kardiovize cohort. This approach differs from traditional *a priori* methods that assess diet based on prior knowledge and scientific evidence^21^. By contrast, principal component analysis (PCA), together with cluster analysis, are the most commonly applied empirical methods, allowing to derive dietary patterns that maximally reflect dietary habits^22^. Next, we performed a cross-sectional analysis to evaluate the association of adherence to each dietary pattern with LV structure, function, and remodelling.

## Methods

### Study design and data collection

Kardiovize Brno 2030 cohort is a prospective study, which enrolled a random sample of 2160 adult residents of the city of Brno (Czech Republic) to assess traditional and novel risk factors for CVD^12–16^. The study protocol was approved by the Ethics Committee of St Anne’s University Hospital, Brno, Czech Republic (reference 2 G/2012) in accordance with the Declaration of Helsinki, and all participants signed an informed consent to participate in the study. To investigate the association of dietary patterns with LVH and remodelling, the current cross-sectional analysis used data from 438 participants with no history of CVD, and with complete assessment of anthropometric, biochemical, echocardiographic and dietary information. Socio-demographic (i.e., age, sex, educational level, marital and employment status) and behavioral (i.e., smoking status and physical activity) characteristics were collected by trained interviewers through structured questionnaires. Educational level was categorized as low (primary education), medium (secondary education), or high (tertiary education). Marital status was categorized into living alone (including single, divorced or widowed) or living in couple (including married and other relationships). Employment status was categorized into employed (including full-time or part-time employment) or unemployed (including retired). Smoking status was self-reported and categorized as current, former or never. Physical activity was assessed using the long form of the International Physical Activity Questionnaire (IPAQ-L) and reported as the Metabolic Equivalent of Task (MET-min/week)^23^.

### Physical examination

Physical examination was performed by trained professionals according to standardized and validated protocols^14–16,24^. In brief, height and weight were measured to the nearest 1 cm and 1 kg, respectively, using a medical digital scale with meter (SECA 799; SECA, GmbH and Co. KG, Hamburg, Germany). BMI was calculated and categorized according to the WHO criteria^25^. Waist circumference was measured to the nearest 1 cm by manual tape measurement to define central obesity according to the WHO criteria^26^. Blood pressure was measured using a mercury sphygmomanometer (Baumanometer, W.A. Baum, Co., Inc., USA), and hypertension was defined as blood pressure ≥140/90 mmHg, or a prior diagnosis or taking antihypertensive drugs. As described elsewhere^6^, biochemical analyses were performed on 12-h fasting blood samples using a Modular SWA P800 analyzer (Roche, Basel, Switzerland). Specifically, total cholesterol, triglycerides and glucose concentrations were measured by the enzymatic colorimetric method (Roche Diagnostics GmbH, Germany), while HDL-cholesterol using the homogeneous method (Sekisui Medical, Japan). LDL-cholesterol concentrations were calculated using the homogeneous method or the Friedewald equation according to triglycerides levels. Hyperlipidemia was defined as having either total cholesterol of ≥5.0 mmol/L, or LDL cholesterol of ≥3 mmol/, or triglycerides of ≥1.7 mmol/L, or taking lipid-lowering drugs. Diabetes mellitus was defined as fasting glucose of ≥7 mmol/L, or a prior diagnosis or taking antidiabetic drugs.

### Echocardiography

Transthoracic echocardiography was performed with a GE-Vingmed Vivid E9 device (GE Vingmed Ultrasound AS, Horten, Norway) using a 1,5–4,6 MHz sector transducer, as described elsewhere^6^. In brief, images were obtained with the patient lying in a left lateral decubitus or supine position, and an ECG signal was recorded and displayed simultaneously. Analyses were performed using the EchoPAC PC software (version 113) and assessed according to the criteria of the American Society of Echocardiography (ASE)^27–29^. RWT was calculated as the ratio of the posterior wall thickness at end-diastole (LVPWd) doubled and left ventricle end-diastolic diameter (LVIDd). LVH was classified as LVMI (i.e. indexed to height^2.7^) >48 g/m^2.7^ for men or >44 g/m^2.7^ for women, according to ASE and the European Association of Cardiovascular Imaging (EACVI) recommendations^30^. LV geometry patterns were classified according to LVMI (i.e. normal or increased) and RWT (i.e. normal or altered). Normal LV geometry was described as RWT <0,42 and LVMI ≤48 g/m^2.7^ for men or ≤44 g/m^2.7^ for women; cLVH was described as RWT >0,42 and LVMI >48 g/m^2.7^ for men or >44 g/m^2.7^ for women; eLVH was described as RWT < 0,42 and LVMI >48 g/m^2.7^ for men or >44 g/m^2.7^ for women and cLVR was described as RWT >0,42 and LVMI ≤ 48 g/m^2.7^ for men or ≤48 g/m^2.7^ for women^27,30^.

### Dietary assessment

Dietary data were collected through a 43-item Food Frequency Questionnaire (FFQ), using the previous week as reference period^12^. During the interview, participants were asked to indicate frequency of consumption classified (seven categories from “almost never” to “six or more times a day”). Standard portion size was attributed to each food item as the age- and sex-specific median food intake obtained from a dietary survey on the national level^31^, which involved age and gender representative sample of the Czech population^32^. Food intakes were calculated by multiplying frequency of consumption by standard serving size, and adjusted for total energy intake using the residual method^33^. To avoid the potential influence of outliers, subjects in the 5th and 95th percentiles of total energy intake were excluded from further analyses. PCA has been widely used in nutritional epidemiology to derive population-dependent dietary patterns. Thus, we classified food items into 31 predefined food groups based on the similarity of nutrient profiles or culinary usage^12^. Next, PCA followed by varimax rotation was performed on energy-adjusted intakes of each predefined food group to derive a posteriori dietary patterns. The number of dietary patterns to retain was defined based on eigenvalues >2.0, Scree plot examination, and interpretability of components. More details on PCA method are reported elsewhere^12,34–36^. Dietary patterns were described based on factor loadings with absolute value ≥0.25. Factor scores were calculated for each dietary pattern by summing the products between observed energy-adjusted food group intakes and their factor loadings, so that higher factor scores indicated higher adherence to dietary patterns and viceversa. To obtain a similar number of participants in each group of adherence, we categorized the adherence to each dietary pattern according to tertile distribution of factor scores as follows: low adherence (1st tertile of factor score), medium adherence (2nd tertile), or high adherence (3rd tertile). In a previous study on the Kardiovize cohort^12^, we confirmed the internal reproducibility of this method by performing separate PCA in two randomly selected subgroups.

### Statistical analyses

All statistical analyses were conducted using SPSS software (version 22.0, SPSS, Chicago, IL). The Kolmogorov-Smirnov test was used to test the normality of continuous variables, and those underlying a skewed distribution were described using median and interquartile range (IQR) and compared using the Kruskal–Wallis test. Spearman’s correlation analysis was used to test correlation between continuous variables. Categorical variables were described using frequency (%) and compared using the Chi-square test. Logistic regression analysis was used to evaluate the association of adherence to dietary patterns with LVH, concentric remodelling and abnormal LV geometry patterns, using low adherence as reference group. Model 1 was adjusted for age, sex, BMI, and waist circumference, while model 2 further adjusted for smoking status, total energy intake, physical activity, diabetes and hypertension. All statistical tests were two-sided, and p values < 0.05 were considered statistically significant.

## Results

### Correlation between food intakes and echocardiographic parameters

**Figure 1:**
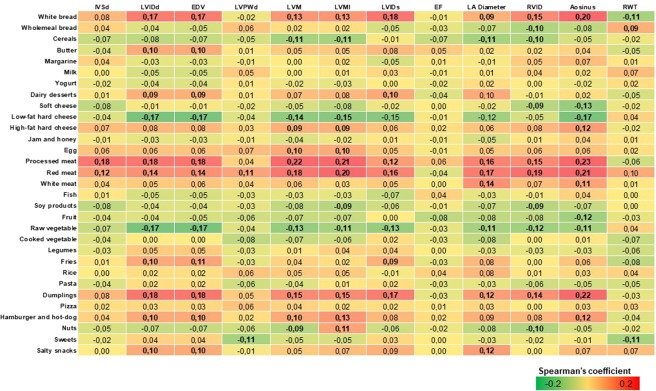
Correlation matrix between food intakes and echocardiographic parameters. Results are reported as Spearman’s correlation coefficient and those with p-value < 0.05 are indicated in bold. Abbreviations: interventricular septum thickness at end-diastole, IVSd; end-diastolic volume, EDV; posterior wall thickness at end-diastole, LVPWd; left ventricle mass, LVM; left ventricle mass indexed to height^2.7^, LVMI; left ventricle end-systolic diameter, LVIDs; ejection fraction, EF; left atrial diameter, LA Diameter; right ventricle diameter, RVID; aortic diameter at the sinus of Valsalva, Aosinus; relative wall thickness, RWT.

The Kardiovize Brno 2030 cohort included 438 participants (59.1% women), aged 25 to 65 years, who satisfied the selection criteria and could be included in the current analysis. In this subsample, we first tested correlations between food intakes and echocardiographic parameters (Fig. 1). In general, we observed that some food groups (e.g. cereals, low-fat cheese, fruit, raw vegetables, and nuts) negatively correlated with echocardiographic parameters associated with LV structure (IVSd, LVIDd, EDV, LVPWd, LVM, LVMI, LVIDs, LA Diameter, RVID, Aosinus, RWT) and function (EF). By contrast, other food groups (e.g. white bread, high-fat cheese, red and processed meat, dumplings, and salty snacks) positively correlated with the same echocardiographic parameters.

### Identification of dietary patterns

**Figure 2:**
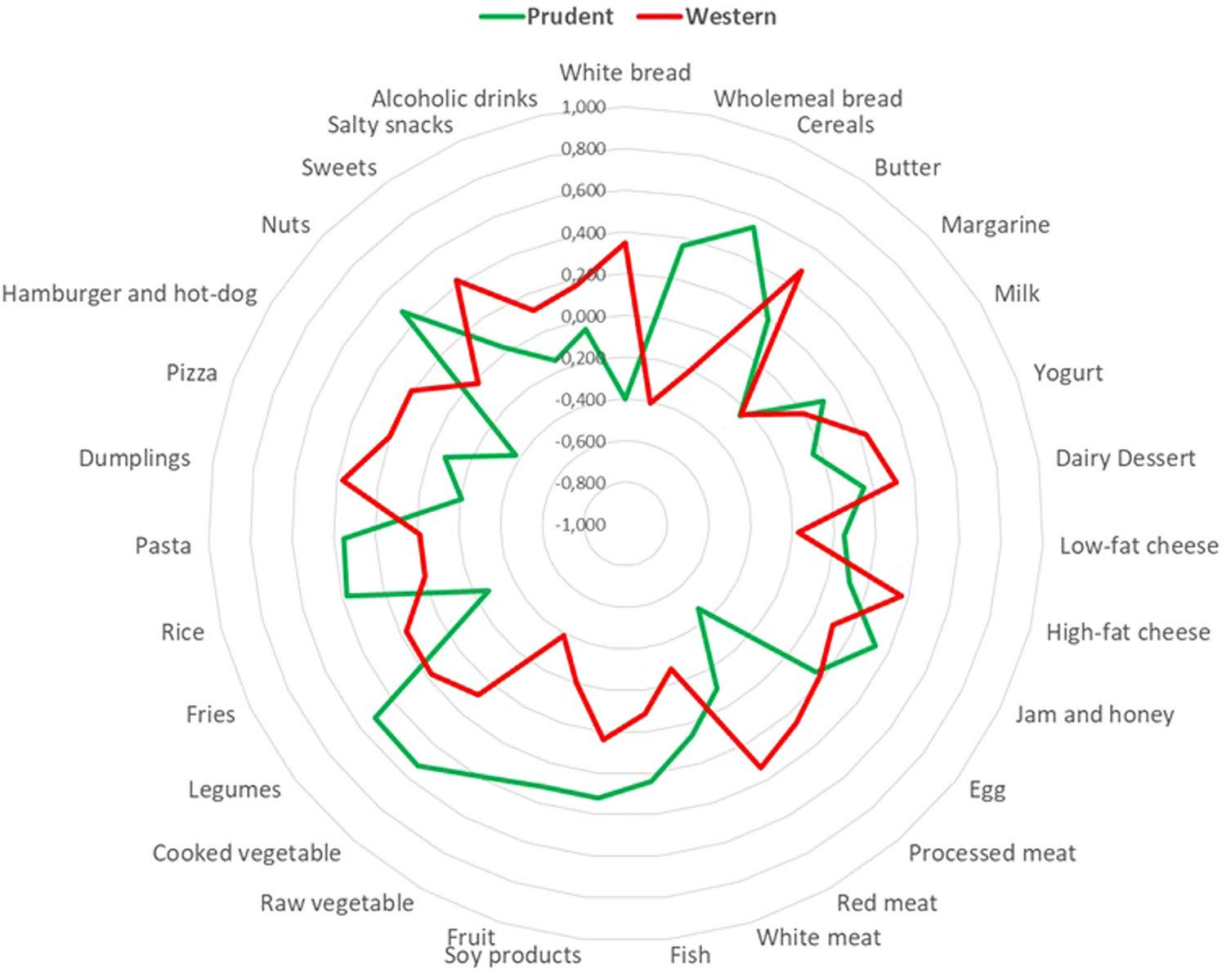
Radar graph of factor loadings characterizing dietary patterns. Red line indicates factor loadings related to the western dietary pattern. Green line indicates factor loadings related to the prudent dietary pattern. Dietary patterns are described based on factor loadings with absolute value ≥0.25.

To address to what extent dietary patters, rather than specific food groups, may affect LV remodelling, we first derived two dietary patterns with eigenvalues ≥2.0, which explained 13.8% of total variance among 31 predefined food groups. Figure 2 shows factor loadings, which can be viewed as the correlation between each food group and dietary pattern. Accordingly, the first dietary pattern - characterized by high intake of whole-meal bread, cereals, jam and honey, soy products, fruit, raw and cooked vegetables, legumes, rice and pasta, and low intake of white bread, processed meat, fries, hamburger and hot-dog - was defined prudent, which was consistent with the well-accepted term used in this field of research^12,34^. Participants with high adherence to the prudent dietary pattern were older, less frequently men, less likely to live alone, and more frequently unemployed and physical active than those with low adherence (Table 1). With respect to cardio-metabolic parameters, participants with high adherence to the prudent dietary pattern exhibited lower waist circumference and triglycerides levels, higher HDL-cholesterol levels, and lower prevalence of obesity and hypertension (Table 1). By contrast, the second dietary pattern - characterized by high intake of white bread, butter, sweets, high-fat cheese, red and processed meat, and dumplings, and low intake of whole-meal bread, low-fat cheese, white meat, and raw vegetables - was named western. Participants with high adherence to the western dietary pattern were younger, more frequently men, and had more total energy intake than those with low adherence (Table 1). With respect to cardio-metabolic parameters, participants with high adherence to the western dietary pattern exhibited higher waist circumference and triglycerides levels, and lower HDL-cholesterol levels (Table 1).

**Table 1:** Characteristics of study population by adherence to dietary patterns.

Characteristics	Prudent				Western				Total
	High adherence (1st tertile)	Medium adherence (2nd tertile)	High adherence (3rd tertile)	p-value	High adherence (1st tertile)	Medium adherence (2nd tertile)	High adherence (3rd tertile)	p-value	
Age, years	45.0 (17.0)	47.0 (18.0)	48.0 (18.0)	**0.014**	50.0 (18.0)	45.5 (17.0)	43.0 (16.0)	**<0.001**	47.0 (19.8)
Sex (% male)	56.4%	54.1%	40.4%	**0.007**	35.7%	48.8%	66.5%	**<0.001**	40.9%
Educational level (% low)	11.9%	8.5%	7.4%	0.560	9.1%	6.8%	11.9%	0.283	10.2%
Marital status (% living alone)	42.6%	35.4%	27.7%	**0.013**	37.7%	39.5%	28.4%	0.064	39.3%
Employment (% unemployed)	4.7%	15.0%	20.2%	**<0.001**	17.4%	12.9%	9.9%	0.117	16.9%
Smoking (% current smokers)	22.2%	22.0%	14.7%	0.213	17.6%	18.1%	23.2%	0.341	20.1%
Total energy intake, kcal	2064 (924)	2108 (882)	2051 (933)	0.193	1910 (836)	2119 (930)	2234 (941)	**<0.001**	1995 (946)
Physical activity, MET-min/week	2570 (3796)	3169 (4483)	3585 (4489)	**0.040**	3205 (4028)	3162 (3515)	2758 (4536)	0.694	3267 (4696)
BMI, Kg/m^2^	25.3 (6.7)	25.0 (5.0)	24.6 (5.7)	0.072	24.7 (6.2)	24.7 (5.8)	25.6 (4.6)	0.243	24.5 (6.0)
Obesity (%)	22.2%	10.2%	8.5%	**0.002**	13.1%	13.6%	14.1%	0.213	13.5%
Waist circumference	90.0 (20.0)	88.0 (16.0)	86.0 (19.0)	**0.035**	86.0 (19.0)	86.0 (19.0)	90.0 (18.0)	**0.009**	85.0 (20.0)
Central Obesity (%)	25.8%	20.3%	22.3%	0.490	25.6%	18.1%	24.6%	0.207	22.2%
Systolic Blood Pressure, mmHg	117.5 (20.0)	117.0 (18.9)	116.8 (19.0)	0.734	118.5 (23.5)	117.3 (16.3)	114.0 (17.0)	0.188	115.5 (18.6)
Diastolic Blood Pressure, mmHg	79.8 (13.1)	79.8 (15.4)	79.0 (11.4)	0.591	80.0 (12.5)	79.8 (14.0)	78.0 (13.0)	0.623	78.8 (13.0)
Hypertension (%)	42.0%	35.6%	26.6%	**0.009**	39.8%	36.2%	28.2%	0.067	35.0%
Fasting Glucose, nmol/l	4.9 (0.6)	4.8 (0.6)	4.9 (0.7)	0.355	4.8 (0.7)	4.8 (0.7)	4.9 (0.7)	0.050	4.8 (0.5)
Diabetes (%)	2.3%	1.1%	0.6%	0.359	1.1%	1.7%	1.1%	0.867	1.3%
Triglycerides, nmol/l	1.1 (0.8)	1.0 (0.8)	0.9 (0.7)	**0.003**	0.9 (0.5)	1.0 (0.9)	1.1 (0.9)	**0.001**	1.0 (0.8)
Total Cholesterol, nmol/l	5.3 (1.5)	5.0 (1.4)	5.0 (1.3)	0.186	5.2 (1.4)	5.1 (1.3)	5.0 (1.5)	0.177	5.1 (1.4)
HDL Cholesterol, nmol/l	1.4 (0.5)	1.5 (0.4)	1.6 (0.5)	**<0.001**	1.6 (0.5)	1.5 (0.5)	1.4 (0.5)	**<0.001**	1.6 (0.6)
LDL Cholesterol, nmol/l	3.1 (1.3)	3.0 (1.2)	3.1 (1.1)	0.360	3.1 (1.3)	3.1 (1.3)	3.2 (1.0)	0.164	3.0 (1.3)
Hyperlipidaemia (%)	42.0%	32.2%	27.1%	0.051	28.4%	33.9%	39.3%	0.110	36.0%

Results are reported as median (Interquartile range), or percentage. Statistical analyses were performed using Chi-square test for bivariate or categorical variables, and Kruskal–Wallis test for continuous variables.

Abbreviations: metabolic equivalent task, MET; body mass index, BMI; high-density lipoprotein, HDL; low-density lipoprotein, LDL.

### Association of dietary patterns with echocardiographic parameters

**Figure 3:**
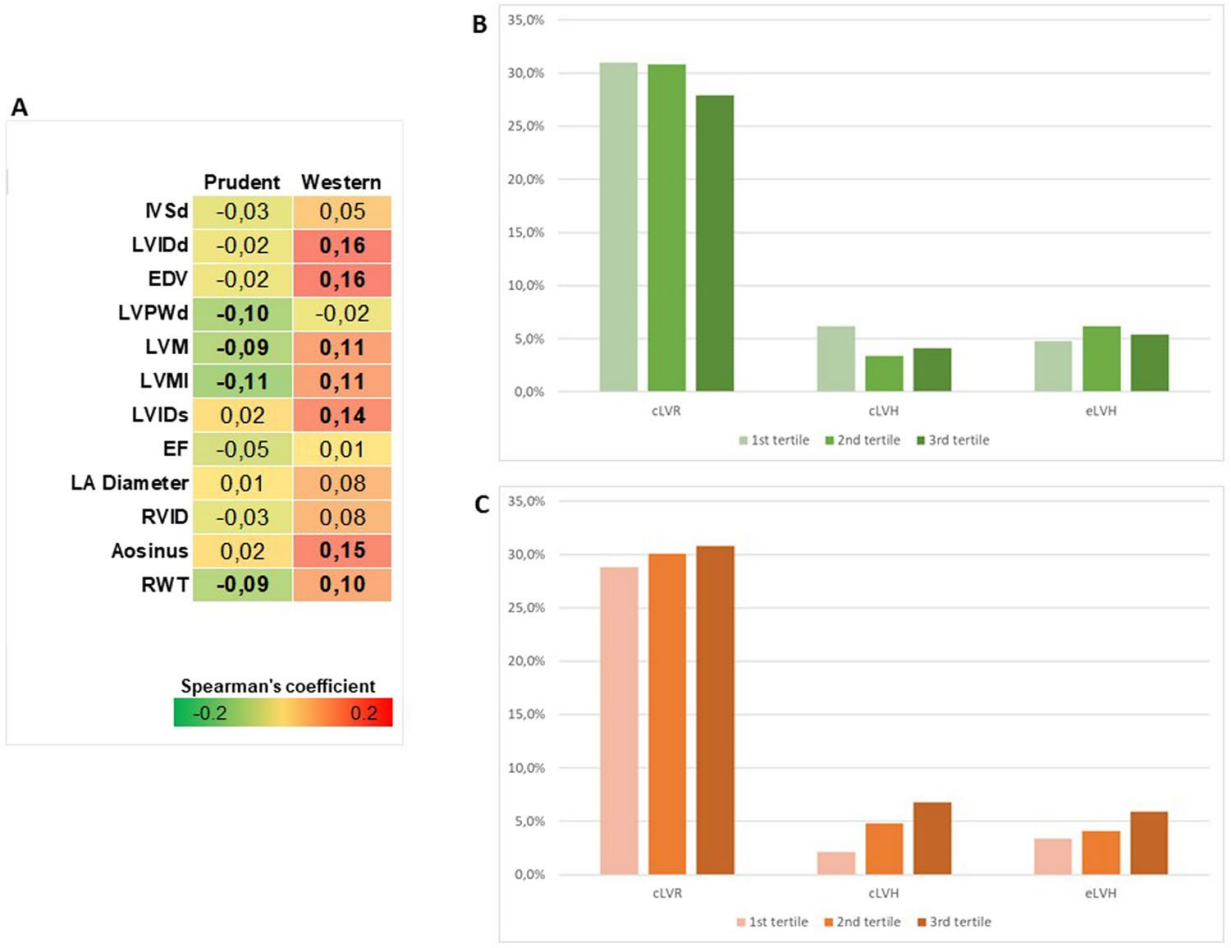
Association of dietary patterns with echocardiographic parameters and left ventricular remodelling. (**A**) Correlation matrix between factor scores and echocardiographic parameters; results are reported as Spearman’s correlation coefficient and those with p-value < 0.05 are indicated in bold. (**B**) Distribution of left ventricular remodelling patterns by adherence to the prudent dietary pattern. (**C**) Distribution of left ventricular remodelling patterns by adherence to the western dietary pattern. Abbreviations: interventricular septum thickness at end-diastole, IVSd; end-diastolic volume, EDV; posterior wall thickness at end-diastole, LVPWd; left ventricle mass, LVM; left ventricle mass indexed to height^2.7^, LVMI; left ventricle end-systolic diameter, LVIDs; ejection fraction, EF; left atrial diameter, LA Diameter; right ventricle diameter, RVID; aortic diameter at the sinus of Valsalva, Aosinus; relative wall thickness, RWT.

We next compared echocardiographic parameters across tertiles of adherence to each dietary pattern (Table 2). While no differences with respect to the prudent dietary pattern were evident, we observed increasing trend of LVID in diastole, EDV, LVMI, LVID in systole, Aosinus, and prevalence of LVH from the bottom to the top tertile of adherence to the western dietary pattern. This was consistent with weak but significant positive correlations between factor score of western dietary pattern and several echocardiographic parameters, including LVM, LVMI and RWT (Fig. 3A). By contrast, factor score of prudent dietary pattern was weakly but negatively correlated with LVM, LVMI and RWT. Since LVMI and RWT allowed to discriminate patients with LV remodelling, we next assessed the relative variations in LV geometry according to adherence to each dietary pattern. Overall, cLVR was the most prevalent abnormal pattern in the whole cohort (29.9%), while either eLVH or cLVH were less frequent (5.5% and 4.6%, respectively). Participants with high adherence to the prudent dietary pattern exhibited lower but not significant prevalence of cLVR and cLVH than those with low adherence (Fig. 3B). By contrast, participants with high adherence to the western pattern exhibited higher but not significant prevalence of cLVR, cLVH, and eLVH than those with low adherence (Fig. 3C). We finally performed logistic regression analyses to determine the association of dietary patterns with LVH, concentric remodelling and specific LV geometry patterns. Although adherence to the prudent dietary pattern did not seem to affect LVH or concentric remodelling in general, it was associated with cLVH. Indeed, compared to low adherence to the prudent pattern, high adherence significantly decreased the odds of cLVH after adjusting for age, sex, BMI, and waist circumference (OR = 0.28, 95% CI = 0.10–0.94; p = 0.030), and further adjusting for physical activity, smoking status, total energy intake, diabetes and hypertension (OR = 0.24, 95% CI = 0.08–0.88; p = 0.031) (Table 3). By contrast, compared to participants with low adherence to the western dietary pattern, those with high adherence were more likely to exhibit LVH (OR = 2.54, 95% CI = 1.09–5.89; p = 0.030) and specifically cLVH (OR = 5.38, 95% CI = 1.17–23.58; p = 0.035; Table 4), after adjusting for age, sex, BMI, waist circumference, physical activity, smoking status, total energy intake, diabetes and hypertension.

**Table 2:** Echocardiographic parameters by adherence to dietary patterns.

Echo parameters	Prudent				Western			
	1st tertile	2nd tertile	3rd tertile	p-value	1st tertile	2nd tertile	3rd tertile	p-value
IVSd	0.9 (0.3)	0.9 (0.1)	1.0 (0.2)	0.829	0.9 (0.3)	1.0 (0.2)	0.9 (0.2)	0.447
LVIDd	4.7 (0.68	4.7 (0.8)	4.7 (0.5)	0.534	4.6 (0.6)	4.7 (0.7)	4.8 (0.7)	**0.002**
EDV	103.5 (39.8)	101.0 (35.5)	101.0 (28.0)	0.568	99.0 (30.0)	103.0 (34.0)	106.5 (36.5)	**0.001**
LVPWd	0.9 (0.1)	0.9 (0.2)	0.9 (0.2)	0.128	0.9 (0.2)	0.9 (0.2)	0.9 (0.2)	0.802
LVM	157.9 (59.7)	156.4 (56.0)	146.9 (55.2)	0.121	147.7 (59.2)	157.5 (58.1)	157.9 (54.4)	0.073
LVMI	37.2 (19.7)	33.0 (17.4)	32.4 (19.9)	0.246	32.5 (18.7)	35.4 (18.2)	38.1 (22.4)	**0.018**
LVIDs	3.1 (0.6)	3.1 (0.5)	3.1(0.5)	0.977	3.0 (0.5)	3.1 (0.5)	3.2 (0.4)	**0.004**
EF	63.0 (8.0)	66.0 (7.0)	63.0 (7.0)	0.148	63.0 (7.0)	64.0 (8.0)	64.0 (7.8)	0.893
LA diameter	3.4 (0.7)	3.6 (0.6)	3.5 (0.7)	0.994	3.6 (0.7)	3.5 (0.7)	3.5 (0.6)	0.131
RVID	3.2 (0.5)	3.2 (0.7)	3.1 (0.6)	0.627	3.1 (0.6)	3.1 (0.6)	3.2 (0.6)	0.227
Aosinus	3.1 (0.7)	3.3 (0.5)	3.2 (0.5)	0.087	3.1 (0.7)	3.2 (0.6)	3.3 (0.4)	**<0.001**
RWT	0.40 (0.10)	0.39 (0.08)	0.39 (0.10)	0.280	0.38 (0.09)	0.40 (0.09)	0.41 (0.09)	0.064
LVH (%)^a^	11.0%	9.6%	9.5%	0.889	6.2%	10.3%	13.7%	**0.039**
Concentric remodelling (%)^b^	37.2%	34.2%	32.0%	0.637	32.2%	35.6%	35.6%	0.777

Results are reported as median (Interquartile range) or percentage. Statistical analysis was performed using Kruskal–Wallis test for continuous variables and Chi-squared test for bivariate variables.

^a^Defined as LVMI >48 g/m^2.7^ for men or >44 g/m^2.7^ for women.

^b^Defined as RWT >0.42.

Abbreviations: interventricular septum thickness at end-diastole, IVSd; end-diastolic volume, EDV; posterior wall thickness at end-diastole, LVPWd; left ventricle mass, LVM; left ventricle mass indexed to height^2.7^, LVMI; left ventricle end-systolic diameter, LVIDs; ejection fraction, EF; left atrial diameter, LA Diameter; right ventricle diameter, RVID; aortic diameter at the sinus of Valsalva, Aosinus; relative wall thickness, RWT; left ventricular hypertrophy, LVH.

**Table 3:** Logistic regression analysis of the association of adherence to the prudent dietary pattern with left ventricular remodelling and hypertrophy.

Regression Model	Prudent pattern	cLVR		cLVH		eLVH	
		OR (95% CI)	p-value	OR (95% CI)	p-value	OR (95% CI)	p-value
Model 1	1^st^ tertile	Ref		Ref		ref	
	2^nd^ tertile	0.94 (0.54–1.54)	0.868	0.48 (0.16–1.42)	0.178	0.85 (0.32–2.26)	0.718
	3^rd^ tertile	0.78 (0.47–1.31)	0.330	0.28 (0.10–0.94)	0.030	0.58 (0.24–1.58)	0.236
	Trend^a^	0.578		0.034		0.509	
Model 2	1^st^ tertile	Ref		Ref		ref	
	2^nd^ tertile	0.99 (0.68–1.73)	0.987	0.58 (0.26–1.12)	0.308	0.92 (0.40–2.44)	0.853
	3^rd^ tertile	0.87 (0.55–1.38)	0.364	0.24 (0.08–0.88)	0.031	0.58 (0.27–1.55)	0.231
	Trend^a^	0.601		0.040		0.482	

^a^p-value for trend. Results are expressed as multivariable-adjusted odds ratio (OR) and 95% confidence interval (CI). Statistical analysis was performed using logistic regression adjusting for age, sex, BMI and waist circumference (Model 1), and further adjusting for physical activity (MET), smoking status, total energy intake, diabetes and hypertension (Model 2).

Abbreviations: concentric left ventricular remodelling, cLVR; concentric left ventricular hypertrophy, cLVH; eccentric left ventricular hypertrophy; reference group, Ref.

**Table 4:** Logistic regression analysis of the association to the western dietary pattern with left ventricular remodelling and hypertrophy.

Regression Model	Western pattern	cLVR		cLVH		eLVH	
		OR (95% CI)	p-value	OR (95% CI)	p-value	OR (95% CI)	p-value
Model 1	1^st^ tertile	Ref		Ref		Ref	
	2^nd^ tertile	0.87 (0.1–1.45)	0.599	3.04 (0.76–12.31)	0.112	0.65 (0.20–2.00)	0.437
	3^rd^ tertile	0.84 (0.48–1.43)	0.512	3.70 (0.92–14.87)	0.062	1.73 (0.65–4.69)	0.300
	Trend^a^	0.838		0.168		0.216	
Model 2	1^st^ tertile	Ref		Ref		Ref	
	2^nd^ tertile	0.94 (0.56–1.54)	0.683	4.63 (0.95–20.65)	0.057	0.71 (0.28–2.03)	0.513
	3^rd^ tertile	0.93 (0.57–1.53)	0.718	5.38 (1.17–23.58)	0.035	1.45 (0.57–4.34)	0.499
	Trend^**a**^	0.912		0.041		0.387	

^a^p-value for trend. Results are expressed as multivariable-adjusted odds ratio (OR) and 95% confidence interval (CI). Statistical analysis was performed using logistic regression adjusting for age, sex, BMI and waist circumference (Model 1), and further adjusting for physical activity (MET), smoking status, total energy intake, diabetes and hypertension (Model 2).

Abbreviations: concentric left ventricular remodelling, cLVR; concentric left ventricular hypertrophy, cLVH; eccentric left ventricular hypertrophy; reference group, Ref.

## Discussion

Our study showed associations between dietary patterns and LV structure, function and remodelling in participants with no history of CVD from the Kardiovize cohort, a randomly selected sample of urban residents of Brno, Czech Republic. Ours, to the best of our knowledge, is the first study to examine the effects of dietary patterns derived using PCA. Indeed, using a dietary pattern approach, rather than the more traditional focus on single nutrients or foods, may be more effective in identifying risk factors for LVR with an immediate relevance for public-health strategies. In this context, factor analysis and PCA, similar in their mathematical basis, have been largely applied to construct a linear function that maximally explains the variation in food intakes of the study population^37,38^. In the present study, we derived a prudent dietary pattern, which was characterized by high intake of whole-meal bread, cereals, jam and honey, soy products, fruit, raw and cooked vegetables, legumes, rice and pasta, and low intake of white bread, processed meat, fries, and hamburger and hot-dog. We demonstrated that, compared with low adherence, high adherence to this dietary pattern was not only favorably associated with low prevalence of obesity and hypertension, but also decreased the odds of cLVH, after adjusting for demographic, behavioral, and clinical covariates. These findings partially confirmed previous evidence that adherence to the Mediterranean diet improved LV structure and function in participants from the Multi-Ethnic Study of Atherosclerosis (MESA) study and the Northern Manhattan study^17,18^. In line with this evidence, in the MESA cohort, the DASH diet - which emphasized consumption of fruits, vegetables, whole grains, poultry, fish, nuts, and low-fat dairy products and minimized consumption of red meat, sweets, and sugar-sweetened beverages - was favorably associated with LV volume, stroke volume, and ejection fraction^19^. Indeed, these dietary patterns improved ventricular filling, which in turn increased end-diastolic filling, LV volumes, stroke volume, and ejection fraction^17^. However, in the MESA study, these findings were not indicative of LVR because there was no association of Mediterranean diet with LV mass-to-volume ratio and LVH^17^, defined as LVM above the population-specific 95th percentile^39^. The choice of method to define LVH for risk prediction and reduction has long been debated reaching no general consensus^11,40^. As previously proposed, the best methods could be LVM normalization to free fat mass or to height^41^. Since free fat mass was not routinely measured in the Kardiovize cohort, we adopted the method based on normalization of LVM to height^2.7^, which is the main factor to contribute to the magnitude of free fat deposition. Differences in the choice of method to define LVH might partially explain controversy between our results and those reported by previous studies. Using PCA, we also derived a western dietary pattern characterized by high intake of white bread, butter, sweets, high-fat cheese, red and processed meat, and dumplings, and low intake of whole-meal bread, low-fat cheese, white meat, and raw vegetables. Adherence to this dietary pattern was associated not only with unhealthy metabolic profile, but also with echocardiographic parameters, including LVM, LVMI and RWT. Accordingly, participants with high adherence to the western dietary pattern were more likely to exhibit LVH, specifically cLVH, than those with low adherence, after adjusting for demographic, behavioral, and clinical covariates. This is consistent with a research on the MESA cohort, which used the reduced rank regression to derive a dietary pattern that maximally explained the variation in metabolic syndrome components. This dietary pattern - characterized by intake of foods with a high glycemic index, high-fat meats, cheeses, processed foods and low intake of vegetables, soy, fruit, green and black tea, low-fat dairy desserts, seeds and nuts, and fish - was unfavorably associated with LV mass and systolic function.

The observed effects of dietary pattern on LV structure, function and remodelling might be attributed to intakes of individual foods. Indeed, we observed that healthy foods, such as cereals, low-fat cheese, fruit, raw vegetables, and nuts, positively affected echocardiographic parameters. By contrast, the same parameters were exacerbated by high-fat, hypercaloric, refined and processed foods. However, from a public health perspective, the identification of protective or deleterious dietary patterns remains the most relevant approach towards development of novel strategies against LVR and LVH. The observed relationships might be also mediated by associations between dietary patterns and several CVD risk factors, especially those related to LVR and LVH. As we previously demonstrated in the whole Kardiovize cohort^12^ and now reaffirmed in current subsample, the western dietary pattern had a deleterious effect on several cardio-metabolic risk factors, while the consumption of a diet rich in cereals, fish, fruit and vegetables was associated with a healthier cardio-metabolic profile. Consistently, previous epidemiological studies demonstrated that intake of healthy foods was associated with more favorable blood pressure^42,43^, insulin sensitivity (38), fasting glucose^42,44^, and lipid profile^42^. More recently, Lara and colleagues demonstrated that adherence to a dietary pattern rich in fried food, organ meats, processed meats, eggs, added fats, and sugar-sweetened beverages was associated with increased risk of heart failure, whereas a plant-based dietary pattern was inversely associated with incident heart failure risk^45^.

Limitations of our study included its cross-sectional design, which did not allow us to understand causality of observed relationships. Moreover, one of the main weaknesses of FFQ is that it relies on memory and on the skills of interviewer. However, using the previous week as reference period could reduce reporting biases due to memory. Additionally, we used a FFQ with standard portion sizes - which did not preclude potential measurement errors and may suffer from inaccuracies - and total variance explained by the PCA-derived dietary patterns was relatively low. To manage potential errors, standard portion sizes were obtained from an individual dietary survey on the national level, and subjects in the 5th and 95th percentiles of total energy intake were excluded. Despite these limitations, food compositions of our data-derived dietary patterns were consistent with those identified by previous studies in different European countries^34,46–49^. However, this approach does not allow to compare the effects of different dietary patterns. Accordingly, we compared and reported findings on the effects of different degrees of adherence to each dietary pattern. Finally, we cannot rule out the possibility of bias from residual confounders that might affect the association of dietary patterns with LV structure, function, and remodelling.

To our knowledge, our study is the first examining associations of PCA-derived dietary patterns with LV structure, function and remodelling. This approach may be more effective in identifying risk factors for LVR, rather than more traditional focus on single foods or a priori dietary pattern. Notably, participants with adherence to a prudent dietary pattern exhibited lower prevalence of obesity, hypertension, and cLVH. In contrast, the adherence to a western dietary pattern worsened their cardio-metabolic profile, which translated into higher prevalence of cLVH. Although our findings may have an immediate relevance for public-health strategies, further large-size prospective studies should be encouraged to better understand the observed association and their causality.

## Data Availability

The datasets analysed during the current study are available from the corresponding author on reasonable request.
